# Reimagining Home Enteral Nutrition Through Artificial Intelligence: A Narrative Review of Clinical, Operational, and Patient-Centered Applications

**DOI:** 10.3390/nu18162613

**Published:** 2026-08-10

**Authors:** Danelle A. Johnson, Edwin Feghali, Osman Mohamed Elfadil, Jithinraj Edakkanambeth Varayil, Manpreet S. Mundi, Ryan T. Hurt

**Affiliations:** 1 Division of Endocrinology, Diabetes, Metabolism, and Nutrition, Mayo Clinic, Rochester, MN 55905, USA; 2 School of Medicine-Wichita, The University of Kansas, Wichita, KS 67214, USA; 3 Department of Family Medicine, Mayo Clinic, Rochester, MN 55905, USA; 4 Division of General Internal Medicine, Mayo Clinic, Rochester, MN 55905, USA

**Keywords:** home enteral nutrition, artificial intelligence, machine learning, large language models, remote monitoring, clinical decision support, precision nutrition

## Abstract

Home enteral nutrition (HEN) is essential for patients with a functioning gastrointestinal tract who cannot meet nutritional needs orally, yet outpatient management remains complex, resource-intensive, and supported by a limited evidence base. Artificial intelligence (AI) may augment HEN care by extending monitoring, education, risk assessment, documentation support, and operational coordination into the home environment. Consistent with the narrative review format, this article uses a pragmatic, transparent synthesis of influential HEN-specific literature, relevant clinical nutrition evidence, and background knowledge from adjacent fields, including home healthcare, chronic disease management, oncology nutrition, telehealth, and software regulation. Direct evidence in established HEN populations remains scarce; therefore, most AI applications should be considered hypotheses or early implementation opportunities rather than proven standards of care. The strongest near-term opportunities are clinician-supervised patient education, symptom triage, adherence support, remote monitoring, and workflow automation. Predictive analytics, smart pumps, and precision enteral prescription tools are promising but require prospective HEN-specific validation, interoperability with electronic health records and home-infusion systems, reimbursement pathways, and governance safeguards. Key barriers include dataset bias, limited external validation, alert fatigue, privacy and regulatory concerns, unclear accountability, digital equity, cost uncertainty, and the risk of dehumanizing care. AI should be viewed as a complement to multidisciplinary HEN expertise. Priorities for the near future include HEN registries, standardized outcomes, prospective validation, pragmatic implementation trials, health-economic evaluation, and transparent oversight that preserves clinician accountability and patient-centered care.

## 1. Background: Home Enteral Nutrition and Artificial Intelligence Foundations

### 1.1. Epidemiology and Standard of Care for Home Enteral Nutrition

Home enteral nutrition (HEN) is essential therapy for patients with a functioning gastrointestinal (GI) tract who are unable to meet nutritional needs by oral intake alone [1]. Enteral nutrition (EN) may be delivered as a commercial formula or blenderized tube feeding through an enteral access device (EAD). It may serve as either short-term or long-term nutrition support depending on the underlying diagnosis, prognosis, and goals of care. The prevalence of HEN in the United States has increased over time, with estimates demonstrating over 440,000 recipients of HEN, yet the evidence base guiding longitudinal outpatient management remains limited [2,3].

Patients on HEN are clinically heterogeneous and frequently medically complex. Optimal care requires coordinated management by a multidisciplinary team that may include a registered dietitian nutritionist RDN, nurse, pharmacist, advanced-practice professional, and physician from the time of feeding tube placement through eventual tube removal when feasible [1]. In practice, however, access to dedicated HEN expertise is variable, and many patients and caregivers must manage formula administration, EAD care, medication delivery, supply logistics and symptom troubleshooting with intermittent clinical contact. This gap is clinically important because HEN-related complications are common; EAD-related complications, metabolic abnormalities, and enteral feeding intolerance (EFI) occur frequently, and complications may lead patients to seek emergency care rather than timely specialty support [1].

Artificial intelligence (AI) may provide a strategy to enhance HEN care by supporting clinicians with technologies such as Machine Learning (ML), natural language processing (NLP), computer vision, smart-device algorithms, and generative AI, including large language models (LLMs), that extend monitoring, patient education, and clinical decision support into the home. AI includes several complementary technologies. Traditional rule-based systems rely on predefined decision rules, whereas ML identifies patterns from data to generate predictions [4,5,6]. Deep learning is a subset of ML that uses multilayer neural networks to model complex relationships [4]. NLP enables computers to analyze and generate human language, whereas computer vision interprets image-based data [4,6]. Generative AI produces new content from learned patterns [7,8]. LLMs are a subset of generative AI trained on large datasets and are primarily used for conversational interactions and information synthesis [9]. Although direct evidence in HEN is limited, findings from clinical nutrition, home parenteral nutrition (HPN), telehealth, and chronic disease self-management suggest that these tools may improve workflow efficiency and support functions to usher in workflow efficiency and prompt a shift toward data-informed models of care [10]. In this narrative review, we assess the current and emerging uses of AI in HEN, consider how these technologies may support clinical decision-making, and discuss the safeguards and research needed to enable safe adoption in clinical practice.

### 1.2. AI Definitions and Taxonomy for Home Enteral Nutrition

AI has continued to revolutionize health care. Across disciplines and various settings, AI tools are used increasingly for documentation and administrative tasks, as well as assistance with diagnostic-related decisions, treatment recommendations, and prognosis prediction [7]. Currently, there is a paucity of data on the implementation of AI in the HEN population. LLMs are readily available generative AI applications that generate human-like responses based on datasets and all other available information to process and synthesize content. ChatGPT (OpenAI) is one such LLM that has demonstrated—in one study that has evaluated GPT-3.5 model—the ability to support clinical decision-making and provide education for managing multiple medical diagnoses [8].

One study examined ChatGPT’s responses compared to those of health care providers in assisting with the care of HPN patients [11]. HPN patients are similar to HEN patients in that they are medically complex and are expected to perform the necessary tasks to administer their primary source of nutrition. ChatGPT has been shown to provide dependable nutrition-related advice and is one of the most reliable LLMs in providing nutrient information about foods. One limitation of ChatGPT is that it struggles to pick up on nuances with individual medical conditions [12,13]. However, among HPN patients, ChatGPT’s responses were preferred over HPN expert clinicians as they expressed more empathy and generated patient-preferred responses to best practices of HPN use, broad medical responses and concerns over certain lifestyle topics. However, ChatGPT had difficulty with topics addressing complex clinical decisions such as lab values, thus validating the need for oversight by expert clinicians [11]. There is an opportunity for exploring this in HEN patients, specifically.

## 2. AI Technologies and Applications in HEN

### 2.1. Screening, Risk Stratification, and Predictive Analytics

HEN is a growing practice, yet complications remain common. In a large prospective cohort of 1600 HEN patients, over half [53.2%] experienced at least one complication, including EFI, EAD-related problems, and electrolyte disturbances [1]. Another study reported that nearly 20% of 30-day hospital readmissions in patients discharged home on EN were directly EN-related [14]. Current follow-up strategies rely on periodic clinic or telehealth visits, intermittent labs, and caregiver-reported symptoms, a reactive approach that frequently identifies problems only after they have escalated. AI may offer the potential to shift this paradigm toward continuous, proactive risk detection in patients already receiving HEN (Table 1).

Malnutrition screening is the first step in identifying patients who need nutrition support. Validated tools such as the Malnutrition Screening Tool (MST), Nutrition Risk Screening 2002 (NRS-2002), and Malnutrition Universal Screening Tool (MUST) are widely recommended [15]. However, these tools have well-documented limitations: they show only moderate validity and reliability, suffer from inconsistent adherence in clinical practice, and were not designed to predict the specific complications that arise in the home setting [16]. Moreover, they provide a static, one-time risk assessment and cannot account for the dynamic changes in a patient’s condition after hospital discharge.

AI, particularly ML, offers the ability to integrate large, multidimensional datasets and generate individualized risk profiles that go beyond what traditional screening tools can achieve [17,18]. One such ML model was externally validated in a retrospective cohort of 106,449 hospitalized adults and demonstrated an AUROC of 0.92 (95% CI, 0.91–0.92) for predicting hospital malnutrition on the first day of admission, outperforming the modified Malnutrition Screening Tool used in clinical practice [19]. Earlier malnutrition identification can lead to decreased hospital length of stay, fewer hospital readmissions and decreased mortality [19]. The continued evolution and implementation of AI tools could bridge the gap in malnutrition screening in the outpatient setting, as malnutrition is not exclusive to the inpatient population. However, it is certainly worth noting that these findings are derived from hospitalized patient cohorts rather than patients receiving HEN. Hence, these studies should be interpreted as evidence extrapolated from adjacent clinical settings rather than validated HEN-specific evidence.

### 2.2. AI Screening, Risk Stratification and Predictive Analytics

ML models integrate longitudinally collected data such as weight trends, EN formula intake logs, symptom reports, and laboratory values to facilitate a continuous nutritional status assessment and flag potential for deterioration before this decline becomes clinically apparent [17,20]. Predictive models for specific HEN-relevant complications already exist: ML models applied to electronic health record data in home healthcare have predicted hospitalization and emergency department visits within a 5-day window, with a time-series Light Gradient Boosting Machine model achieving an F1 score of 0.84. Key predictors included visit frequency, length of nurse-patient encounters, and clinical note content extracted via natural language processing, which alone improved predictive performance by up to 16.6% [21]. For HEN-specific complications, deep learning models such as NutriSighT have predicted underfeeding with AUROCs of 0.76–0.81, while aspiration risk prediction models have achieved AUROCs exceeding 0.92 using variables such as consciousness level, comorbidity burden, and aspiration history that are readily available in the home setting [20,22]. When incorporated into telehealth and remote-monitoring systems used currently for HEN follow-up, these models may support earlier identification of patients whose evolving clinical and nutritional data suggest an increased risk of feeding intolerance, aspiration, or subsequent hospitalization. However, all existing predictive models were developed in hospital or general home healthcare populations, and prospective validation in established HEN cohorts is essential before clinical deployment [23]. It is also important to note that many of these are retrospective studies from single centers, and this may overestimate the performance of the AI models and limit their generalizability. Whether these models maintain similar accuracy when applied to the heterogeneous HEN population remains unknown.

### 2.3. Remote Monitoring, Smart Devices, and Telehealth Integration

At the present time, there remain many opportunities for AI integration into the care of HEN patients to help prevent common complications and provide ongoing support. Though there are efforts to learn more about AI integration into the nutrition support practice in general, there is a lack of longitudinal data and outcomes to validate developed AI-driven nutrition therapies.

One area that is being explored in patients receiving EN in the intensive care unit that could also carry over into HEN is predicting EFI. In a retrospective study of 487 patients admitted to the ICU, an ML model incorporating close to 100 clinical data points demonstrated excellent predictive performance for enteral feeding tolerance [24]. Though more research is warranted, a similar model could be employed by a HEN team to guide decisions regarding HEN care, such as formula or EAD type to prevent adverse effects, thus setting up the patient for the highest chance of success. Similarly, predicting other complications surrounding EADs, such as infection, would also be advantageous.

Second, HEN patients require a high amount of support and education to perform necessary tasks appropriately, preferably from experts in HEN. When living with an EAD, this causes changes to typical daily life, and the need to know how to manage issues that may arise to avoid hospitalization. Some investigation into the perceptions of HEN patients and their caregivers reinforces the need for continued support from healthcare professionals, not only routinely but also in urgent situations, as well as improved education and a desire for personalized recommendations [25,26]. This signals a need for an evidence-based AI tool readily available for HEN patients and created by HEN experts. There is evidence that providing such a tool for cancer patients has revealed compelling improvement in patients’ symptom management and quality of life [27].

Aside from the potential for AI-based apps to assist patients and machine learning models to predict issues with tolerance of EN, there remains a potential for the integration of AI into existing tools or supplies utilized to administer the formula. Specifically, enteral feeding pumps could be revolutionized to automate nutrition delivery to make up for missed nutrition or a deficit in a patient’s prescribed goal compared to what they have received in a day. Although no validated tools are currently in use for HEN patients, a nutrition management system was developed for intensive care unit (ICU) patients receiving nasogastric tube (NGT) feedings. The smART+ system involves an NGT with impedance sensors that detect episodes of reflux and tube movement, which can then pause feedings and inflate a balloon to prevent formula from reaching the oropharynx and resulting aspiration, in instances of significant reflux. It also determines missing nutrition provisions when feeds are paused for either reflux or other indications and can then make up for this nutrition over the course of 24 h. This was shown to be effective in improving feeding efficiency compared with standard-of-care feeding protocols [28]. However, these findings were generated among in-patients admitted to the ICU and have not been evaluated in the HEN population. In HEN patients, however, similar technology could assist, especially when patients are not able to achieve their nutritional goals due to circumstances that limit their ability to administer feedings (examples including but not limited to other medical procedures or appointments, a daily schedule different from the normal, travel, etc.).

### 2.4. Precision Nutrition, Workflow Automation, and Operational Logistics

The application of AI in precision nutrition has presented multiple opportunities to advance the personalization of nutrition recommendations to manage and prevent disease, as well as to optimize overall health and wellness. An increasing amount of research has been conducted in this space, with the majority of focus on diseases with diet implications, including diabetes, cardiovascular disease, and cancer. Other conditions that have been investigated include neurodegenerative diseases, eating disorders, gastrointestinal disorders, and mental health disorders. Conventional AI methods are the most commonly tested in the existing body of research, which function on logic and previously defined rules, such as lab values or other pieces of patient data, to produce personalized nutrition recommendations [29]. If conventional AI methods have been implemented in the aforementioned disease states and conditions, they could also potentially be utilized in individualizing recommendations for HEN patients, given that HEN patients often carry multiple medical diagnoses. For example, decisions regarding EAD type and formula selection are typically shared decisions between patients and clinicians; however, with additional information, perhaps AI’s input could help to prevent future complications. Also, precision nutrition could be employed by RDNs who design enteral regimens to determine macronutrient goals and possibly determine the formula best suited for patients.

Anecdotally, it can be challenging for HEN patients to comply with their nutrition recommendations, as everyday life poses barriers to fulfilling goals when relying on alternative nutrition. Given that patients also lack access to experienced HEN clinicians, this could pose a significant nutritional risk to patients. Another area in which AI technology could assist is attempting to fill this gap, with AI-based coaching. Studies have shown that coaching interventions via AI can significantly improve adherence to diet and physical activity recommendations [30]. Specialty AI apps could be developed to serve the HEN patient population in this context, and to help mitigate issues as they arise—this ultimately could lead to improved patient outcomes and patient satisfaction.

Aside from directly impacting patients, AI has benefited clinicians and enabled workflow automation. This has resulted in significant time savings, with a recent study demonstrating decreases in documentation time and time spent reviewing records. This then increased the ability to conduct additional visits [31]. This data was collected among physicians and advanced practice clinicians; however, these results in theory could translate to RDNs and other HEN clinicians as well. At this time, there is significant variance in the adoption and accessibility of this technology among clinicians other than physicians and advanced practice providers.

## 3. Clinical Outcomes, Real-World Evidence, and Patient Perspectives

### 3.1. Clinical Outcomes

In clinical nutrition as a whole, AI and ML models have shown the ability to improve clinical outcomes and assist with clinical decision-making. Though research has mostly focused on areas including oncology nutrition or inpatient nutrition, work to validate this potential in HEN patients is needed. Studies have reported the use of ML algorithms to predict EFI, diarrhea, and refeeding hypophosphatemia, enabling earlier intervention and potentially preventing complications [17]. These predictive models integrate gastrointestinal symptoms, demographic parameters, and severity scores to anticipate feeding success and guide nutritional therapy decisions [32]. In the home setting, AI-driven decision support has the potential to personalize EN prescriptions, optimize nutrient delivery, and reduce complications such as aspiration, EAD dysfunction, and malnutrition-related hospital readmissions [18]. Furthermore, ML-based malnutrition screening tools have shown promise in identifying at-risk patients earlier and more accurately than traditional screening instruments, facilitating the timely initiation of appropriate nutritional therapy [19,33].

### 3.2. Real-World Evidence

Real-world implementation of AI in nutritional care remains in its early stages but is growing. A national implementation study of an AI-based virtual dietitian [“Ina”] enrolled 3310 cancer patients across all 50 US states between 2019 and 2023. The platform provided conversational, text message-based nutritional guidance derived from over 100,000 expert-curated interventions and clinical guidelines. Engagement was high, with a median user retention of 8.8 months, and 84% of users reported applying the AI-generated advice to guide their dietary habits [27]. A separate evaluation of large language model-based AI assistants demonstrated that these tools can reliably replicate expert-level clinical reasoning in complex nutrition support cases, suggesting a role for AI-enabled e-consultation to bridge gaps in nutrition expertise among non-specialist physicians [30]. Although these implementation studies support the feasibility of AI-enabled nutritional care, they provide only indirect evidence for HEN because they were conducted primarily in oncology or general nutrition populations. Telemedicine-based HEN services have also shown real-world feasibility, with revisit rates increasing from 25.8% to 35.8% when digital platforms were integrated into follow-up care [34].

### 3.3. Patient Perspectives

Although patients generally view AI favorably, many continue to express reservations regarding its role in clinical care. Patients’ literacy with technology and AI needs to be considered, as does access to devices to perform related tasks. In a multicenter survey of 230 gastroenterology patients, 61% reported that AI could augment physician care, whereas 93% believed physicians should remain responsible for final clinical decisions, and 74% wanted disclosure when AI was involved in their care [35]. Concerns about reliability [60%], data privacy [47%], and healthcare costs [45%] were prevalent, particularly among racial minorities and individuals with lower education or income [35]. Patients utilizing an AI-based virtual RDN reported overwhelmingly positive satisfaction and perceived benefit: 94% were satisfied with the platform, 98% found the guidance helpful, 82% reported improved quality of life, and 88% noted improved symptom management [27]. In the HEN population specifically, qualitative evidence indicates that patients and caregivers who have access to adequate support systems and coping mechanisms view HEN as a worthwhile experience, whereas those with limited support report predominantly negative experiences [36]. The incorporation of AI tools into HEN care may help bridge this divide by offering continuous, accessible, and personalized support. At the same time, concerns about diminished personal interaction emphasize the need for AI’s role to strengthen and augment, rather than replace, relationships between clinicians and patients [18]. Additionally, patient perspectives regarding AI implementation in HEN, including the design of technology, usability, and thresholds for altering pertinent clinical markers, will be of utmost value.

## 4. Ethical, Regulatory, and Economic Considerations

### 4.1. Ethical Considerations

Integrating AI technologies into HEN raises concerns regarding algorithmic bias, accountability, data privacy, and the decrease in human connection or rapport. AI models trained on datasets that do not take the needs of underserved populations into consideration may produce less accurate recommendations, which could lead to exacerbated health disparities [18]. When AI-generated nutritional recommendations are erroneous with the potential to lead to harm (for example, AI dietary plans containing contraindicated foods), the delineation of responsibility among developers, clinicians, and institutions remains unclear [18]. Data privacy concerns are amplified by the continuous collection of sensitive health information required by AI systems [37]. Lastly, AI must augment rather than replace the clinician–patient relationship, particularly in the HEN population, where inadequate support already contributes to negative patient experiences [18].

### 4.2. Interoperability and Workflow Integration

Interoperability is a practical prerequisite for HEN AI. Clinicians need data from EHRs, patient portals, laboratory systems, telehealth platforms, home-infusion vendors, pump or sensor devices, and patient-reported outcome tools. Data should flow into existing workflows rather than requiring staff to monitor multiple disconnected dashboards. Relevant implementation decisions include which data fields are standardized, how patient-generated health data enter the EHR, who is responsible for reviewing alerts, how urgent findings are escalated, how AI-generated documentation is labeled, and how corrections are made when the AI output is wrong. Alignment with health information exchange and interoperability frameworks will be necessary for scalable deployment.

### 4.3. Regulatory Considerations

Regulatory standards specific to AI in clinical nutrition are evolving. In the United States, the Food and Drug Administration’s regulations primarily oversee AI technologies classified under the Software as a Medical Device framework, although many nutrition-focused applications, including chatbots and dietary-planning software, may fall outside regulatory oversight under the 21st Century Cures Act [38]. In contrast, the European Union AI Act (2024) establishes a broader, risk-based framework for mandating transparency and human oversight for high-risk clinical applications [38]. A life cycle management approach with continuous real-world performance monitoring has been advocated to address the evolving nature of AI algorithms [38,39].

### 4.4. Economic Considerations

Economic evidence for AI in HEN is limited. Telehealth-delivered nutrition interventions appear cost-effective, with mobile health modalities demonstrating the most favorable profiles [40]. AI-driven tools may reduce costs through automated malnutrition screening, fewer hospitalizations, and optimized nutritional prescriptions [17]. However, a systematic review of health economic evaluations of medical AI found that key cost categories, including implementation and operational costs, were frequently omitted, and nearly all studies (98%) concluded cost-effectiveness, raising concerns about publication bias and methodological rigor [41].

## 5. Data Source, Pragmatic Approach, and Evidence Limitations

### 5.1. Data Source

This article was designed as a narrative review rather than a systematic review, scoping review, or meta-analysis. The distinction is intentional. Systematic reviews are optimal for narrowly framed questions with a sufficiently mature and homogeneous evidence base, whereas narrative reviews are well suited to broad, emerging, interdisciplinary topics that require interpretation, critique, conceptual synthesis, and clinical contextualization. Published methodological discussions emphasize that narrative and systematic reviews serve complementary purposes, and that narrative reviews may draw on purposively selected influential literature, methodological diversity, and expert background knowledge when the rationale, scope, and interpretive boundaries are made transparent [42,43,44,45,46]. Accordingly, the objective of this review was not to produce an exhaustive or reproducible inventory of every AI or digital-health study relevant to nutrition support. Rather, the aim was to synthesize clinically important HEN workflows, identify where direct evidence exists, and distinguish plausible opportunities extrapolated from adjacent fields from applications ready for HEN implementation.

Literature was identified through pragmatic searches of PubMed/MEDLINE, Scopus, and Google Scholar conducted between April and May 2026. Representative search terms included combinations of home enteral nutrition, enteral nutrition, artificial intelligence, machine learning, deep learning, large language models, clinical decision support, remote monitoring, telehealth, nutrition support, and precision nutrition. Reference lists of relevant review articles and key primary studies were also examined to identify additional publications of clinical relevance. We prioritized HEN- and EN-specific and clinically relevant sources, but included adjacent fields when AI applications made sense for HEN. We excluded reports without clinical relevance or enough detail. Instead of formal meta-analysis or risk-of-bias tools, we summarized key study features and limitations to fit the narrative style.

### 5.2. Evidence Limitations

The narrative design of this review should be considered when interpreting its conclusions. The review intentionally emphasizes conceptual integration, clinical context, and research prioritization rather than exhaustive evidence retrieval or effect-size estimation. Source selection may therefore be influenced by author expertise, citation visibility, and publication availability. To mitigate this limitation, we have described the pragmatic source-identification approach, distinguished HEN-specific evidence from adjacent evidence, and avoided claims that AI effectiveness has been established in HEN when only extrapolated data is available (Table 2).

The current evidence base has several important limitations. First, direct AI studies in established HEN cohorts are scarce. Second, much of the evidence comes from single-center, retrospective, proof-of-concept, or adjacent-setting studies. Third, predictive models often lack prospective external validation, calibration reporting, decision–curve analysis, or evidence that alerts improve clinical outcomes. Fourth, data completeness differs substantially between hospitals and homes; home data may be intermittent, patient-reported, or unavailable to the EHR. Fifth, AI evaluations often emphasize positive performance metrics while underreporting failures, maintenance costs, alert burden, workflow disruption, and subgroup inequities.

Publication bias is also likely. Positive AI results are more likely to be published than unsuccessful model-development efforts, and economic evaluations of medical AI frequently omit implementation and operational costs while reporting favorable cost-effectiveness [41]. These limitations justify a restrained interpretation: AI in HEN is promising, but current evidence is insufficient to support broad unsupervised deployment or autonomous regimen adjustment.

## 6. Future Directions and Research Roadmap

Future research should move beyond proof-of-concept applications and prioritize prospective, HEN-specific validations of AI tools in diverse cohorts. Multi-center registries that integrate EHR data, EAD characteristics, formula prescriptions, pump-delivery data, weight trends, laboratory values, patient-reported symptoms, caregiver inputs, and supply-chain information are needed to develop models that predict clinically meaningful outcomes such as EFI, EAD dislodgement or clogging, site infection, or emergency department visits and hospital readmissions. Pragmatic trials should evaluate whether AI-enabled monitoring, coaching, and alerts improve outcomes compared with standard telehealth or usual HEN follow-up, while also measuring clinician workload, cost-effectiveness, and patient trust. Development of these AI models should include input from patients, caregivers, HEN clinicians, home infusion companies, payers, as well as regulatory experts to ensure that the tools are usable, equitable and aligned with real-world workflows. Finally, future studies must address model transparency, bias across age, race, language, geography, diagnosis, socioeconomic status, protection of health information, and the risk of hallucinations before unsupervised deployment in HEN care.

## 7. Conclusions

HEN remains a target for innovation because patients and caregivers manage complex nutrition therapy at home with limited access to specialized support, and AI could strengthen care by integrating symptoms, intake, weight trends, laboratory values, device data, and clinical documentation into earlier risk recognition and more individualized support. In the near term, AI is best viewed as a clinician-supervised adjunct that improves access and timeliness through patient and caregiver education, reminders, adherence support, symptom triage, and guidance on when to contact the HEN team. AI’s potential is two-fold because it can also assist clinicians with malnutrition screening, complication prediction, regimen optimization, and documentation. Predictive analytics, remote monitoring, smart devices, and generative AI may help identify patients needing earlier outreach, detect missed feedings or EAD-related concerns, and reduce administrative burden, but they should not be a substitute for multidisciplinary HEN expertise. The current evidence base remains insufficient for broad, unsupervised AI deployment in HEN because most data is derived from adjacent fields. Future work should prospectively validate AI tools in diverse HEN populations, evaluate clinical and patient-centered outcomes, and establish safe workflows for escalation, oversight, and documentation. In conclusion, current evidence suggests that AI could support a more individualized approach to HEN management. However, realizing this will require continued rigorous validation, transparent governance, privacy protection, bias monitoring, continued clinician oversight in CDM, and ongoing attention to AI performance and safety. Pragmatic trials should compare AI-enabled HEN workflows with usual care or standard telehealth while measuring clinical outcomes, patient experience, caregiver burden, clinician workload, safety events, equity, and cost. Given the rapid evolution of AI in healthcare, the next 5 years should focus on translating promising AI applications into clinically validated HEN tools through prospective studies, implementation research, and multidisciplinary collaboration, ensuring that HEN care continues to evolve while providing cutting-edge, patient-centered care for this medically complex population.

## Figures and Tables

**Table 1:** Potential AI applications for HEN patients.

AI Application	What AI Can Do for HEN Patients	Potential Data Inputs or Tools	Potential Value	Key Safeguards/Limitations
**Risk stratification and predictive analytics**	Identify patients at higher risk for underfeeding, dehydration, electrolyte abnormalities, aspiration, EFI, emergency visits, or readmission.	EHR diagnoses, prior admissions, weight trends, intake logs, labs, medications, symptoms, EAD type, and caregiver reports.	Enables earlier outreach and prioritizes limited HEN team resources toward patients most likely to deteriorate.	Requires HEN-specific validation; avoid opaque risk scores without clinician review.
**Remote monitoring and automated alerts**	Track changes between visits and alert the care team when a patient’s trajectory suggests worsening tolerance, inadequate delivery, or clinical decline.	Connected scales, symptom surveys, pump-delivery records, telehealth platforms, laboratory interfaces, and patient portals.	Moves care from reactive troubleshooting to proactive monitoring and may reduce preventable escalation.	False alarms and missed alerts are possible. Alert fatigue and escalation workflows must be managed.
**Patient and caregiver education**	Provide accessible, plain-language guidance on tube flushing, formula administration, medication timing, troubleshooting, storage, travel, and red-flag symptoms.	Clinician-approved chatbot, app-based education modules, multilingual content, and health-literacy adaptation.	Improves self-management confidence and provides support outside clinic hours.	Content must be expert-curated and should direct urgent symptoms to clinicians or emergency care.
**Symptom triage and escalation support**	Help patients decide whether symptoms such as vomiting, diarrhea, constipation, leakage, pain, fever, clogging, or respiratory concerns require self-care, clinic contact, or urgent evaluation.	Structured symptom checker, patient-reported outcomes, photos when appropriate, and clinician triage protocols.	May reduce delays in care and avoid unnecessary emergency visits when issues can be managed safely by the HEN team.	Must not replace clinical judgment. High-risk symptoms need conservative escalation.
**Smart pumps and sensor-enabled devices**	Measure delivered versus prescribed feeding volume, identify interruptions, compensate for missed nutrition within prescribed limits, and potentially detect reflux or tube movement.	Enteral pumps, impedance sensors, flow sensors, device logs, and programmable algorithms.	May improve feeding efficiency and help patients meet nutrition goals despite daily-life interruptions.	Device algorithms require safety testing, manual override, and clear clinician-defined parameters.
**Precision enteral prescription**	Support individualized formula selection, feeding schedule design, fluid targets, macronutrient distribution, and adjustments for comorbidities or tolerance.	Nutrition assessment, diagnoses, labs, medications, renal or glycemic status, GI symptoms, allergies, preferences, and blenderized feeding considerations.	Helps RDNs and clinicians tailor regimens and anticipate complications before they occur.	Recommendations require RDN/clinician approval. AI may miss nuance or contraindications.
**Adherence support and AI coaching**	Send reminders, track formula intake, identify barriers to completing feeds, reinforce hydration/flush goals, and provide behaviorally tailored coaching.	Mobile app, text messaging, intake logs, schedule preferences, caregiver inputs, and missed-feed patterns.	Supports consistency and may improve nutrition adequacy, quality of life, and patient satisfaction.	Tools should minimize burden and be accessible to patients with limited technology access or low health literacy.
**Tube-site and device complication detection**	Assist with early recognition of peristomal redness, leakage, skin breakdown, infection risk, tube dislodgement, or clogging patterns.	Patient-submitted photos, structured questionnaires, device history, nursing notes, computer vision or NLP.	Promotes earlier intervention for common EAD complications.	Image quality, privacy, bias across skin tones, and clinician confirmation are critical.
**Clinician workflow automation**	Draft notes, summarize patient messages, identify overdue labs or follow-up, generate education materials, and support documentation or prior authorization.	EHR data, telehealth notes, patient portal messages, templates, and guideline-based prompts.	Reduces administrative burden and frees clinicians to focus on complex patient care.	All outputs need human review. PHI protections and institutional policies are essential.
**Supply and operational logistics**	Predict formula and supply needs, flag delivery interruptions, support reordering, and identify patients at risk of running out of formula or tube supplies.	Prescription volume, pump usage, supply orders, shipping records, and patient-reported inventory.	Prevents therapy interruptions and reduces patient/caregiver stress.	Requires integration with vendors and safeguards against inequitable access or coverage barriers.
**Quality improvement and research**	Create HEN registries, identify complication patterns, evaluate outcomes, and support the development of HEN-specific prediction models.	De-identified clinical datasets, patient-reported outcomes, claims, device data, and telehealth encounters.	Builds the evidence base needed for safer, validated AI tools in HEN.	Needs governance, consent/IRB consideration, bias monitoring, and transparent model reporting.

**Table 2:** Summary of the current evidence supporting AI applications relevant to HEN.

Study	Population/Setting	Study Design	AI Method	Clinical Application	Key Findings	Major Limitations	Evidence Category	Level of Evidence
**Bernstein et al. (2025)** [19]	Hospitalized adults	External validation study	Machine learning	Malnutrition screening	ML model identified patients at nutritional risk earlier than traditional screening (AUROC 0.92)	Inpatient population; not validated in HEN	Evidence extrapolated from adjacent settings	Moderate
**Topaz et al. (2025)** [21]	Home healthcare	Retrospective cohort	LightGBM + NLP	Hospitalization prediction	Predicted hospitalization within 5 days (F1 = 0.84)	Retrospective; not HEN-specific	Evidence extrapolated from adjacent settings	Moderate
**Jangda et al. (2025)** [20] **Zhang et al. (2024)** [22]	Severe acute pancreatitis	Predictive model	Machine learning	Aspiration risk prediction	AUROC >0.92	ICU population only	Evidence extrapolated from adjacent settings	Low
**Wang et al. (2025)** [24]	ICU enteral nutrition	Predictive model	Machine learning	Feeding intolerance prediction	High predictive performance	Single clinical setting; requires HEN validation	Evidence extrapolated from adjacent settings	Low
**Kagan et al. (2023)** [28]	ICU enteral nutrition	Randomized clinical trial	Smart sensor-enabled feeding system	Smart feeding pumps	Improved feeding efficiency	Hospital prototype; not home setting	Conceptual opportunity	Low
**Buchan et al. (2024)** [27]	Oncology	National implementation study	AI virtual dietitian	Patient education & symptom management	High engagement and satisfaction	Oncology population	Evidence extrapolated from adjacent settings	Moderate
**Maher et al. (2020)** [30]	Community adults	Proof-of-concept trial	AI virtual coach	Adherence support	Improved diet and activity adherence	General population	Conceptual opportunity	Low
**Rotenstein et al. (2026)** [31]	Physicians & APPs	Multicenter implementation study	Ambient AI	Workflow automation	Reduced documentation burden	Not nutrition-specific	Evidence extrapolated from adjacent settings	Moderate
**Hurt et al. (2026)** [47]	Nutrition support cases	Comparative evaluation	Large language models	Clinical decision support	Expert-level reasoning in selected cases	No patient outcomes	Conceptual opportunity	Low
**Barrera et al. (2026)** [11]	Home parenteral nutrition	Comparative evaluation	ChatGPT	Patient education	High patient preference for empathy	HPN rather than HEN	Evidence extrapolated from adjacent settings	Low

## Data Availability

No new data was created or analyzed in this study. Data sharing is not applicable to this article.
